# Predicting Functional Outcome after Stroke: The Influence of Neglect on Basic Activities in Daily Living

**DOI:** 10.3389/fnhum.2013.00182

**Published:** 2013-05-09

**Authors:** Tanja Nijboer, Ingrid van de Port, Vera Schepers, Marcel Post, Anne Visser-Meily

**Affiliations:** ^1^Experimental Psychology, Helmholtz Institute, Utrecht UniversityUtrecht, Netherlands; ^2^Rudolf Magnus Institute of Neuroscience, Center of Excellence for Rehabilitation Medicine, University Medical Center Utrecht and De Hoogstraat RehabilitationUtrecht, Netherlands; ^3^Revant RevalidatiecentrumBreda, Netherlands

**Keywords:** stroke, neglect, recovery, ADL

## Abstract

One prominent deficit resulting from stroke is visuo-spatial neglect, which has been associated with slower and more attenuated recovery patterns of sensory-motor impairment as well as limitations in activities of daily living (ADL). The aim of the current study was to further specify the relationship between neglect and recovery of different domains of ADL. One hundred eighty four patients were assessed with the Functional Independence Measure in the first week of inpatient rehabilitation, and again at 6, 12, and 36 months post-stroke. On average, neglect patients scored significantly lower on Self-care, Transfers, and Locomotion compared to non-neglect patients, but these differences became smaller with progress of time. Overall, no differences between groups were found for Sphincter control and Cognition. Patients with more severe neglect scored significantly lower on Self-care and Transfers compared to patients with mild neglect. During rehabilitation, it would be of importance to test for independence in ADL domains in neglect in order to define realistic treatment goals. The current findings could be taken into account in early multidisciplinary intervention planning in the sub-acute phase, to optimize regaining ADL.

## Introduction

One prominent deficit resulting from stroke is visuo-spatial neglect, commonly referred to as neglect; about 25–30% of all stroke patients show impaired or lost awareness for events and (visual, auditory, and/or tactile) stimuli located at the side opposite of the brain lesion (Appelros et al., 2002; Buxbaum et al., 2004). Neglect can result from a lesion to either hemisphere, but is more severe and enduring after right hemisphere damage (Stone et al., 1993). The time course of spontaneous neurological recovery of neglect shows a natural logistic curve up to the first 12–14 weeks post-stroke, after which neglect severity becomes invariant (Nijboer et al., 2012). Neglect has been associated with slower and more attenuated recovery patterns of sensory-motor impairment (Katz et al., 1999) as well as limitations in activities of daily living (ADL) (Katz et al., 1999; Cherney et al., 2001; Di Monaco et al., 2011; Verhoeven et al., 2011) compared to non-neglect patients. None of the previous studies, however, differentiated between the different domains of ADL, whereas there is general consensus that some of these domains are more complex (e.g., Self-care, Transfers, Locomotion) than others (e.g., bowel management) (Granger et al., 1993; Grimby et al., 1996). Additionally, skills that easily allow for compensation strategies (e.g., grooming), improve earlier compared to more complex skills (e.g., dressing and climbing stairs) (Kwakkel and Kollen, 2013).

The aim of the current study was to further specify the relationship between neglect and recovery of different domains of ADL. Knowledge about factors that determine the final outcome in terms of post-stroke activities is important for early stroke management, in order to set suitable rehabilitation goals, enable early discharge planning, and psycho-education (Kwakkel and Kollen, 2013). One of the most widely used functional outcomes measures in rehabilitation facilities is the Functional Independence Measure (FIM), which measures degree of disability. Performance on the five domains of the FIM (i.e., Self-care, Sphincter control, Transfers, Locomotion, and Cognition) were compared between neglect and non-neglect patients in a repeated measures design up to 3 years post-stroke. Additionally, the relation between neglect severity and functional independence was investigated, as strong associations between severity of neurological deficits and final basic ADL outcomes have been described (Kwakkel and Kollen, 2013).

## Materials and Methods

### Participants

The “Functional Prognostication and disability study on stroke” (FuPro-stroke) database was used for the current study. The aim of FuPro-stroke was twofold: first, to determine which functional outcome measures are most effective in a stroke population; and second, to investigate prognostic factors of functional outcome and recovery up to 3 years post-stroke onset. In FuPro-stroke, 318 patients were selected from stroke patients consecutively admitted to four Dutch rehabilitation centers for an inpatient rehabilitation program in the period April 2000–July 2002. The inclusion criteria were: (1) first-ever stroke, as revealed by CT or MRI; (2) a one-sided supratentorial lesion; (3) age above 18; and (4) written or verbal informed consent. Exclusion criteria for the FuPro-stroke were: (1) disabling comorbidity [pre-stroke Barthel Index (BI) below 18 (range 0–20)]; (2) premorbid inability to speak Dutch. Exclusion criteria for the present study were: (1) subarachnoid hemorrhage (*n* = 34); (2) no letter cancelation at start of the study (*n* = 100).

### Procedure

Patients were included at the start of rehabilitation. Informed consent was obtained. Personal and stroke characteristics were recorded at the first assessment. The scoring of ADL independency and neglect was assessed in the first week of inpatient rehabilitation, and again at 6, 12, and 36 months post-stroke. The study was approved by the Ethics Review Boards of the University Medical Center Utrecht and all participating rehabilitation centers.

### Outcome measures

The FIM (Linacre et al., 1994; Marshall et al., 1999; Schepers et al., 2006) consists of 18 items assessing level of independence at 5 domains: Self-care [i.e., eating, grooming, bathing, dressing (upper and lower body), toileting], Sphincter control (i.e., bladder and bowel management), Transfers (i.e., bed/chair/wheelchair, toilet, tub/shower), Locomotion (i.e., walk/wheelchair, stairs), and Cognition (i.e., comprehension, expression, social interaction, problem solving, and memory). Each item is scored on a seven-point Likert scale, and the score indicates the amount of observed assistance required to perform each item (1, total assistance, 7, total independence), resulting in a final summed score ranging from 18 up to 126.

Additionally, the patient’s medical record was reviewed. The following *admission* to rehabilitation data were captured: age, gender, time post-stroke, hemisphere and subtype of stroke, BI, Motricity Index (MI), Center of Epidemiologic Studies Depression Scale (CES-D), Mini Mental State Examination (MMSE), and sensory deficit in the arm as determined by the Thumb Finding Test (TFT).

The BI (Collin et al., 1988) measures the extent to which stroke patients can function independently in their ADL (i.e., feeding, bathing, grooming, dressing, bowel and bladder control, toileting, chair transfer, ambulation, and stair climbing). Scores range from 0 (completely dependent) up to 20 (completely independent).

The MI (Collin and Wade, 1990) was used to determine the motor functions. There are three items for the arms (i.e., pinch grip, elbow flexion, shoulder abduction) as well as three items for the legs (i.e., ankle dorsiflexion, knee extension, hip flexion). Scores range from 0 (no activity, paralysis) up to 33 [maximum (normal) muscle force] for each dimension, with a maximum total score of 100.

The CES-D (Shinar et al., 1986; Parikh et al., 1988) was used to determine the magnitude of depressive symptomatology. Scores range from 0 (no depressive symptoms) up to 60 (many depressive symptoms). It investigates mood over the past 7 days.

Cognitive status was measured with the MMSE (Folstein et al., 1975). It is a 30-point questionnaire used for screening orientation, memory, attention, calculation, language, and construction functions. Scores vary from 0 (severe cognitive impairments) up to 30 (no cognitive impairments). A score of less than 24 is considered as cognitive impairment.

In the TFT (Kalra and Crome, 1993; Rieck and Moreland, 2005), the patient is asked to find his thumb with his unaffected hand, while the affected arm supported in front and eyes are closed. Scores vary from 0 (unable) up to 3 (no deficit).

The Letter Cancelation Test (LCT, Lezak, 1995) was used to categorize patients as neglect or non-neglect. In the LCT, patients need to cancel O’s among other letters to demonstrate presence and severity of neglect. Patients were requested to cross all O’s on a sheet of A4 paper containing 20 O’s on the left side and 20 O’s on the right, among 425 distractor letters in total. Both target and distractor letters were arranged in random order throughout the page. The difference in number of crossed letters on the contralesional and ipsilesional side was used to indicate neglect [i.e., an asymmetry of at least two omissions^1^ between contralesional and ipsilesional sides (Kelley and Kovacs, 1986)] and hence, categorize patients as neglect or non-neglect. Severity of neglect was indicated by the magnitude of this asymmetry.

### Statistical analyses

Demographics and stroke characteristics of the neglect and no-neglect patients were compared using Mann–Whitney *U* tests.

The extent of recovery of dependency for functional activities explained by time was estimated using random coefficient analysis with MLWin (Rasbash et al., 2009a,b,c). The advantages of using random coefficient analysis in this case are, first, the explicit “time” variable and second, the efficiency when number of time-dependent measures across individuals varies. As such, information about change within an individual as well as across individuals will be taken into account. Observed differences in change across individuals can be associated with individual characteristics (i.e., important predictors of change over time) (Singer and Willet, 2003).

The iterative generalized least-squares (IGLS) was used to estimate the regression coefficient (Singer and Willet, 2003). Regression coefficients were calculated for the association between outcome (FIM domains: Self-care, Sphincter control, Transfers, Locomotion, and Cognition) and neglect at admission and time, corrected for motor impairment (MI), sensory deficits (TFT), dependence (BI), and magnitude of depressive symptomatology (CES-D) at admission, to certify that potential differences between groups are attributable to neglect and not to other group differences. In addition, interaction terms (neglect × time) were fitted to determine if the post-stroke relationship between neglect at admission (with non-neglect as reference) and outcome was dependent upon the time of measurement.

Additionally, regression coefficients were calculated (neglect patients only) for the association between outcome (FIM domains: Self-care, Sphincter control, Transfers, Locomotion, and Cognition) and neglect severity (i.e., magnitude of asymmetry in left versus right sided omissions on the LCT).

The Wald-test was used to obtain *p*-values for the regression coefficients (Twisk, 2006). For all tests, a two-tailed significance level of 0.05 was used.

## Results

### Demographic and stroke characteristics

In the present aim, 184 patients (mean age: 57.42, SD: 11.09) were included from the original FuPro-stroke database. In general, patients were relatively young and infarctions were more frequent than hemorrhages. Neglect was present at admission in 28.80%. An overview of all demographics and stroke characteristics of the neglect and no-neglect patients is given in Table 1. The groups did not differ with respect to age, gender, time post-stroke, and cognitive impairment. In line with literature, the brain lesion was located in the right hemisphere in most of the neglect patients, whereas this was more equally distributed in the non-neglect patients. Overall, neglect patients showed more sensory deficits, were more impaired in motor functions for both upper and lower extremities and more dependent in ADL at start of the study compared to non-neglect patients, as measured with the TFT, MI, and BI respectively. Furthermore, neglect patients showed more depressive symptoms compared to non-neglect patients.

**Table 1:** **Demographical and stroke characteristics per group (neglect versus non-neglect) at admission**.

Clinical variables	Neglect (SD)	Non-neglect (SD)	Statistics
Group size	53	131	
Age in years	55.5 (10.29)	58.1 (11.33)	*U* = 3846, *Z* = −1.362, *p* = 0.173
Gender (female)	47.2%	35.1%	*U* = 3053, *Z* = −1.517, *p* = 0.129
Time post-stroke in days	56.1 (29.84)	47.6 (20.31)	*U* = 4530, *Z* = −1.247, *p* = 0.212
Hemisphere of stroke (*R*)	88.7%	51.9%	*U* = 2195, *Z* = −4.65, *p* < 0.001
Subtype of stroke			*U* = 2503, *Z* = −3.21, *p* = 0.001
Cortical ischemic (%)	73.6	51.0	
Subcortical ischemic (%)	18.9	28.2	
Intracerebral hemorrhage (%)	7.5	19.8	
BI (0–20)	10.5 (4.2)	13.2 (4.3)	*U* = 1174, *Z* = −2.97, *p* = 0.003
MI UE (0–100)	32.6 (31.1)	58.8 (28.2)	*U* = 1842, *Z* = −4.94, *p* < 0.001
MI LE (0–100)	47.4 (29.9)	58.7 (23.3)	*U* = 2689, *Z* = −2.33, *p* = 0.020
MI total (0–100)	40.0 (28.6)	58.7 (23.3)	*U* = 2150, *Z* = −3.99, *p* < 0.001
CES-D (0–41)	17.3 (9.4)	12.2 (9.3)	*U* = 2218, *Z* = −3.46, *p* = 0.001
MMSE (0–30)	25.6 (2.8)	26.3 (2.6)	*U* = 2883, *Z* = −1.63, *p* = 0.103
Sensory deficit (TFT)			*U* = 2276, *Z* = −3.838, *p* < 0.001
Problem (%)	63.5	36.3	

*BI, Barthel Index; MI, Motricity Index; UE, upper extremities; LE, lower extremities; CES-D, Center of Epidemiologic Studies Depression Scale; MMSE, Mini Mental State Examination; TFT, Thumb Finding Test*.

### Random coefficient analysis

For Self-care, neglect patients scored approximately four points lower at start compared to non-neglect patients, and with each subsequent measurement, this difference decreased with approximately one point (Table 2). For Transfers, neglect patients scored approximately three points lower compared to non-neglect patients, and with each subsequent measurement this difference decreased with approximately one point. Finally, for Locomotion, neglect patients scored approximately two points lower compared to non-neglect patients, and with each subsequent measurement this difference decreased with approximately one point. No differences in time-dependent patterns of recovery were found for Sphincter control and Cognition.

**Table 2:** **Regression coefficients, confidence intervals (CI), and level of significance for the analysis of time-dependency of recovery between the neglect and non-neglect of the dimensions of the Functional Independence Measure (Self-care, Sphincter control, Transfers, Locomotion, and Cognition), corrected for motor impairment (Motricity Index), sensory deficits (Thumb Finding Test), dependence (Barthel Index), and magnitude of depressive symptomatology (CES-D) at admission**.

Task	β value	CI	*P*-value
Self-care			
Neglect	3.79	1.79 to 5.79	<0.001
Time	1.73	1.18 to 2.28	<0.001
Neglect × time	−0.92	−1.57 to −0.28	0.005
Sphincter control			
Neglect	0.11	−0.59 to 0.81	0.764
Time	0.37	0.17 to 0.56	<0.001
Neglect × time	−0.15	−0.38 to 0.08	0.198
Transfer			
Neglect	3.11	1.85 to 4.36	<0.001
Time	1.83	1.46 to 2.21	<0.001
Neglect × time	−1.01	−1.46 to −0.58	<0.001
Locomotion			
Neglect	2.16	1.00 to 3.33	<0.001
Time	1.71	1.36 to 2.06	<0.001
Neglect × time	−0.70	−1.11 to −0.30	0.001
Cognition			
Neglect	0.03	−0.95 to 1.01	0.947
Time	−0.62	−0.88 to −0.37	<0.001
Neglect × time	0.02	−0.28 to 0.32	0.912

### Relation between severity of neglect and ADL

This analysis was performed with neglect patients only. On average, neglect patients showed an asymmetry of 7.62 (SD = 4.16; asymmetry range: 3–19 omissions) omissions on the left versus right side. Patients with more severe neglect scored significantly lower on Self-care and Transfers. No relation between neglect severity and Sphincter Control, Locomotion, and Cognition was found (see Table 3). There was a positive relation between time and all levels of the FIM; with each subsequent measurement, independence on all levels increased (see Table 3). There were no significant interactions between neglect severity and time for any of the levels of the FIM (no modification of the effects; overall, *p* > 0.172), hence, the interaction term was removed from the model.

**Table 3:** **Bivariate regression coefficients, confidence intervals (CI), and level of significance for the analysis of time-dependency of recovery of the dimensions of the Functional Independence Measure (Self-care, Sphincter control, Transfers, Locomotion, and Cognition) as a function of neglect severity (for neglect patients only)**.

Task	β value	CI	*P*-value
Self-care
Severity	−0.506	−0.748 to −0.271	<0.001
Time	1.59	0.70 to 2.48	<0.001
Sphincter control
Severity	−0.060	−0.127 to 0.007	0.078
Time	0.327	0.07 to 0.58	0.012
Transfer
Severity	−0.181	−0.344 to −0.018	0.029
Time	1.83	1.25 to 2.41	<0.001
Locomotion
Severity	−0.104	−0.245 to 0.037	0.149
Time	1.59	1.09 to 2.10	<0.001
Cognition
Severity	0.017	−0.071 to 0.105	0.706
Time	−0.67	−1.00 to −0.34	<0.001

## Discussion

The aim of the current study was to investigate the relation between neglect and recovery patterns of Self-care, Sphincter control, Transfers, Locomotion, and Cognition up to 3 years post-stroke. Results indicated markedly lower scores for patients with neglect on the Self-care, Transfers, and Locomotion scales of the FIM, compared to non-neglect patients at start of the study. These differences decreased with progress of time. For Sphincter control and Cognition, similar scores and time-dependent recovery patterns were found for both groups. Additionally, patients with more severe neglect were more dependent for Self-care and Transfers, but no relation between neglect severity and Sphincter control, Locomotion, and Cognition was found. There was also no relation between neglect severity and time-dependent recovery for any of the levels of the FIM.

Earlier studies also compared ADL performance between neglect and non-neglect patients, yet did not differentiate between different domains of ADL. For example, Cherney et al. (2001) and Katz et al. (1999) found that FIM Motor total scores were significantly lower for neglect patients compared to non-neglect patients. In these studies, patients were tested three times: at admission to a rehabilitation facility, at discharge, and either 3 (Cherney et al., 2001) or 6 months after discharge (Katz et al., 1999). Even though, in both these studies, the initial performance of neglect patients was lower for the Motor items, the results are largely in line with our results; neglect patients scored significantly lower compared to non-neglect patients up to 6 months after discharge. A major strength of the current study compared to the other two studies is that measurements were fixed in times, rather than using a relative moment (i.e., discharge), minimizing variation due to differences in the time elapsed since stroke. Additionally, the current results specified that the patterns of recovery differed for the functional domains of the FIM.

In both the current study as well as the study of Cherney et al. (2001), no differences were found for FIM Cognition scores. Katz et al. (1999), however, did find significant differences between neglect and non-neglect patients with respect to Cognition scores. Katz et al. (1999) showed that patients with severe neglect had lower scores on Cognition items of the FIM compared to patients with less severe neglect. We did not find such a relation between neglect severity and cognition. This discrepancy between studies might be explained by the level of cognitive function at start of study. Here, patients were only included when performance on the LCT was available at start of the study. As such, patients with other cognitive impairments (e.g., language problems) restricting performance on the LCT were excluded. MMSE scores for both neglect and non-neglect groups in the current study were fairly high and might explain the confined influence of neglect on cognitive functions. It is important to note, that the MMSE is a short and broad screening list and the Cognition part of the FIM is an observation scale and as such do not give a full and detailed measure of cognitive performance like when using neuropsychological or experimental tests. It might be that differences between groups would have appeared when using tests with a strong time component, either in duration (e.g., sustained attention versus “rapid” changes) or *ad hoc* decision making in a dynamic environment.

Further examination of demographical and stroke characteristics indicates that, at admission, neglect patients showed more depressive symptoms compared to non-neglect patients. This is in line with the results of Nys et al. (2006) who found that among all cognitive disorders, neglect was the greatest risk for depressive symptoms in the long term. Additionally, neglect has been negatively associated with life satisfaction 1 year post-stroke (Verhoeven et al., 2011).

For skill acquisition, it is important to make a distinction between *restitution* of function (i.e., regaining the ability to perform a given task through the same pre-stroke pattern of activation, Levin et al., 2009) and *substitution* of function (i.e., regaining the ability to perform a given task, but not necessarily through the same pre-stroke pattern of activation, Levin et al., 2009). The former is related to neurological recovery (Krakauer et al., 2012) within the first months post-stroke (Kwakkel et al., 2004; Nijboer et al., 2012), whereas the latter is related to compensatory responses (Krakauer et al., 2012), which are likely to account for recovery after 3 months post-stroke (Kwakkel et al., 2004; Nijboer et al., 2012). As the first follow-up measurement was done 6 months post-stroke, no distinction can be made between restitution and substitution of function in the first few months post-stroke. The question therefore remains whether neglect has a negative influence on spontaneous recovery of functions in the first months post-stroke.

A second possible limitation is ceiling effects, which may be responsible for a relatively long period of stability in recovery (Kwakkel and Kollen, 2013). As such it may be that a difference between groups in magnitude or pattern of recovery may exist, yet the scale will be unable to capture it. With for example the Frenchay Activities Index (Pedersen et al., 1997; Schepers et al., 2006), extended ADL, which require initiative from the patients, are measured. The limitation is that this index cannot be used during the admission to a rehabilitation center, but might be of value during follow-up.

Finally, it is important to note that all patients included in this study received inpatient rehabilitation after hospitalization, which might impede the generalizability. In general, patients referred to inpatient rehabilitation are relatively young and moderately disabled. We did not, however, find a relationship between age and neglect, suggesting that the relatively young age of our sample age does not limit the generalizability of our results.

In conclusion, neglect has a negative influence on functional independence in Self-care, Transfers, and Locomotion, especially in the sub-acute phase. During rehabilitation, it would be of importance to test for *independence in ADL* in neglect in order to define realistic treatment goals. The current findings could be taken into account in early multidisciplinary intervention planning in the sub-acute phase, to optimize regaining ADL.

## Conflict of Interest Statement

The authors declare that the research was conducted in the absence of any commercial or financial relationships that could be construed as a potential conflict of interest.

## Footnotes

^1^One might argue that this cut-off value is rather liberal. Therefore we also grouped patients with a less liberal asymmetry (4); this did not change the results. Therefore, we chose to keep the asymmetry of 2 as criterion for neglect, in line with the norms of the test.
